# Prenatal Diagnosis of a Feingold Syndrome Pregnancy Complicated with Severe Preeclampsia: A Report of a Challenging Case

**DOI:** 10.3390/genes17010054

**Published:** 2026-01-02

**Authors:** Athina A. Samara, Paraskevas Perros, Antonios Koutras, Michel B. Janho, Emmanuil Manolakos, Nikoletta Daponte, Apostolos C. Ziogas, Antonios Garas, Chara Skentou, Sotirios Sotiriou

**Affiliations:** 1 Department of Embryology, Faculty of Medicine, University of Thessaly, Mezourlo, 41110 Larissa, Greece; 2 Department of Obstetrics and Gynecology, Faculty of Medicine, University of Thessaly, Mezourlo, 41110 Larissa, Greece; 3 Department of Obstetrics and Gynecology, Alexandra Maternity Hospital of Athens, National Kapodistrian University of Athens, Lourou 4, 11528 Athens, Greece; paris_per@yahoo.gr (P.P.);; 4 Access to Genome, Michalacopoulou 139, 11528 Athens, Greece; 5 Department of Obstetrics and Gynecology, Faculty of Medicine, University of Ioannina, S. Niarhou, 45500 Ioannina, Greece

**Keywords:** microcephaly, short stature, bilateral clinodactyly, intellectual impairment, gastrointestinal disorders, Feingold syndrome

## Abstract

Feingold syndrome (FS) is a rare congenital disorder with an autosomal dominant inheritance pattern. Two distinct subtypes are recognized based on their molecular pathology: FS type 1 (FS1) and FS type 2 (FS2). Both types share skeletal anomalies such as microcephaly, brachymesophalangia, and clinodactyly; however, gastrointestinal atresia is unique to FS1. Herein, we report a rare prenatal diagnosis of FS1 in a female fetus. The second-trimester ultrasound revealed bilateral clinodactyly and fetal microcephaly, and the subsequent molecular karyotyping identified a ~342 kb deletion at 2p24.3 encompassing the *MYCN* gene, confirming the diagnosis. The same deletion was detected in the father, verifying the hereditary pattern. The pregnancy was also complicated by preeclampsia and fetal growth restriction, leading to preterm caesarean delivery at 33 + 3 weeks of gestation. The neonate had microcephaly and clinodactyly but no gastrointestinal defects. In conclusion, high clinical suspicion aroused by identifying ultrasound features of FS can lead to early prenatal diagnosis via molecular karyotyping. Detecting accompanying gastrointestinal disorders that require early operation is crucial for the prognosis, genetic counseling, and prenatal management of the affected families.

## 1. Introduction

In 1975, Murray Feingold reported the first case of Feingold syndrome (FS), where three members in a single family (mother, son, and grandson) presented with microcephaly, hand abnormalities, tracheoesophageal fistula, and duodenal atresia. He also stated that the three individuals had normal intelligence levels [1]. The inheritance model of FS is autosomal dominant. De novo mutations where the parents did not show any dysmorphic features have been reported [2]. FS is regarded as an uncommon syndrome whose precise prevalence remains undetermined given the limited number of reports in the literature, which consist of small patient cohorts and case reports.

The autosomal dominant inheritance of FS has been demonstrated in many families over several generations [3]. Although the condition is autosomal dominant, more than 60% of patients are female [1]. Two distinct subtypes are recognized based on their molecular pathology: FS type 1 (FS1) and FS type 2 (FS2). The exact incidence of FS1 remains unknown; however, since it was first described in 1975, more than 200 cases have been reported, mainly as isolated case reports or small case series [2].

The syndrome is characterized by microcephaly, short stature, brachymesophalangia, thumb hypoplasia, toe syndactyly, and intellectual impairment. The overall pattern of malformations in FS shows considerable overlap with the VATER/VACTERL group of congenital syndromes (vertebral defects, anal atresia (imperforate anus), cardiac defects, tracheoesophageal fistula, esophageal atresia, renal anomalies, and limb abnormalities) [4]. Hand abnormalities, microcephaly, short and/or narrow palpebral fissures, broad nasal bridge, anteverted nostrils, ear abnormalities, micrognathia, and intestinal atresia comprise the most prevalent characteristics of FS. Dysmorphic features, intellectual disability, and other organ anomalies such as cardiac defects and renal abnormalities, are less frequently described [2]. FS1 is differentiated from FS2 by the presence of gastrointestinal atresia in the former [4,5,6]. Due to the variety of clinical presentation, the syndrome has also been described as oculo–duodeno–esophageal–digital (ODED) syndrome, microcephaly–oculo–digitoesophageal–duodenal syndrome, and microcephaly–mesobrachyphalangy–tracheoesophageal fistula (MMT) syndrome [7]. Moreover, some cases share variable features included in distinct ‘‘microcephaly–digital abnormalities–normal intelligence’’ conditions [8]. Herein, we aim to present a rare case of a female fetus with a prenatal diagnosis of FS1.

## 2. Case Presentation

A 35-year-old primiparous Caucasian female (gravida 0) with a spontaneous singleton pregnancy presented to a fetomaternal specialist physician for a routine second-trimester scan. Her body mass index was normal and her medical and gynecological histories were unremarkable. During the first-trimester ultrasound, a singleton gestation was confirmed with, normal modified probabilities for trisomies and low probabilities for preeclampsia and preterm delivery.

**Figure 1:**
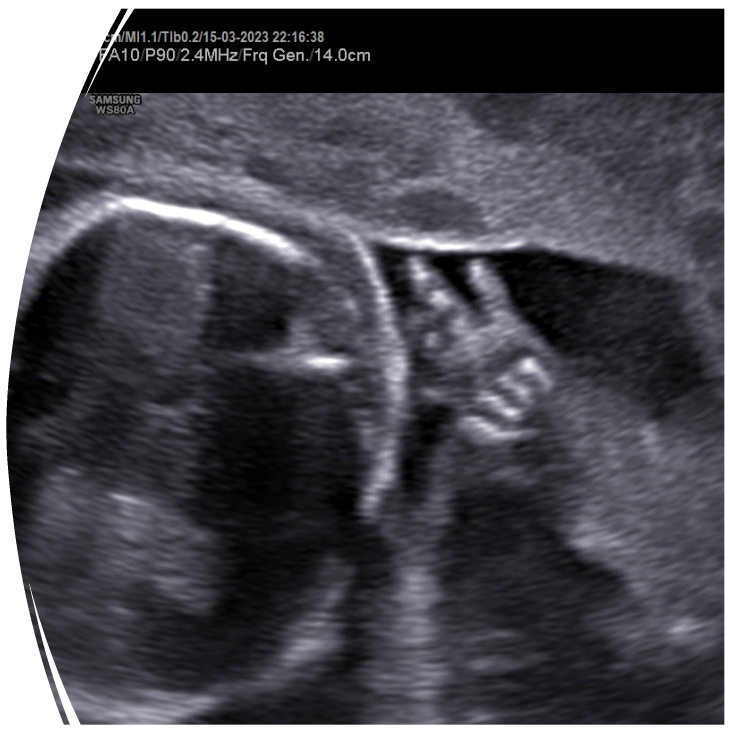
Βilateral clinodactyly was noted in the second-trimester ultrasound.

**Figure 2:**
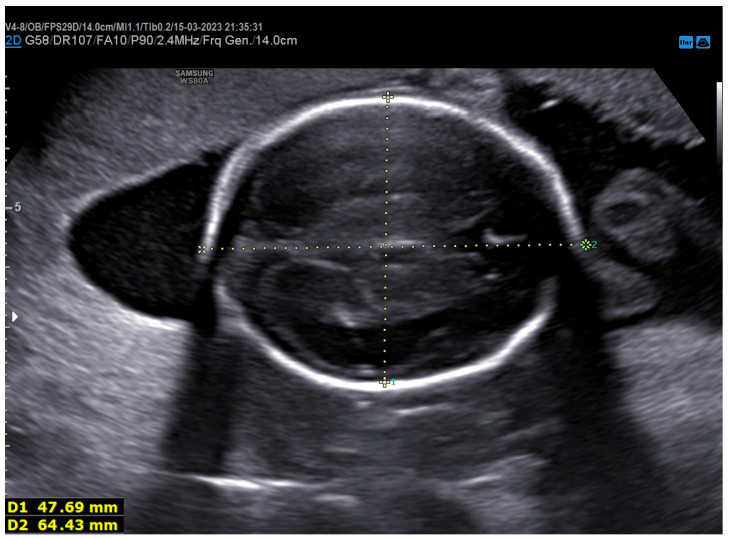
Head growth was under the 5th percentile for gestational age in the second-trimester ultrasound.

**Figure 3:**
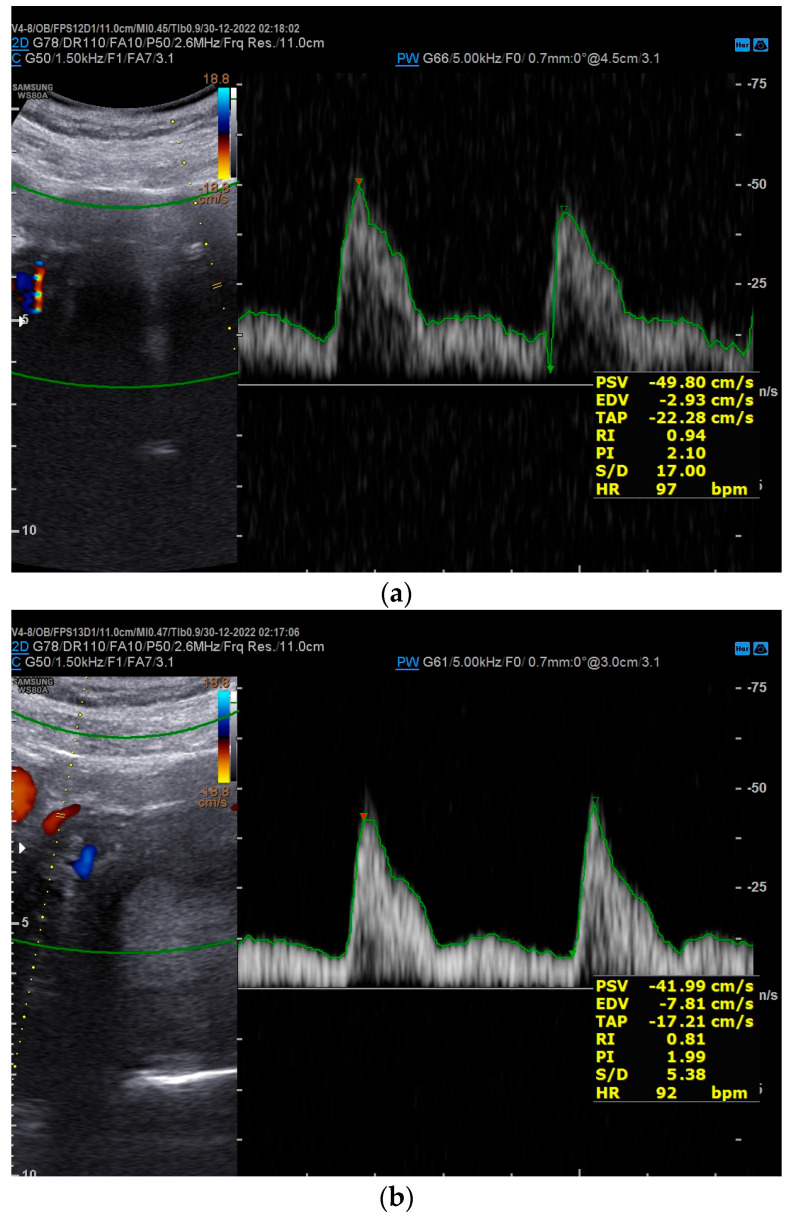
(**a**,**b**) Increased resistance in the maternal vessels, both with nodules, in second-trimester ultrasound.

In the scheduled check for the second-trimester ultrasound, bilateral clinodactyly was detected (Figure 1). Furthermore, fetal development was consistent with the estimated gestational age, with normal sonographic measurements apart from the head measurements—biparietal distance (BPD) and head circumference (HC)—which were under the 5th percentile for gestational age (Figure 2). Increased resistance was observed in the maternal vessels, and both had nodules (Figure 3a,b).

Based on the abnormal prenatal ultrasound findings, invasive prenatal genetic testing was recommended, and amniocentesis was performed. Chromosomal microarray analysis (CMA (Thermo Fisher Scientific, Waltham, MA, USA) ) using a high-resolution genome-wide copy number detection platform (array-CGH) identified a heterozygous ~342 kb deletion at chromosome 2p24.3 (genomic coordinates chr2:15,768,340–16,111,204, GRCh37). This copy number variant encompasses four OMIM-listed genes: *DDX1* (OMIM 601257), *MYCNUT* (OMIM 615968), *MYCNOS* (OMIM 605374), and *MYCN* (OMIM 164840). The deletion results in the loss of one copy of *MYCN*, leading to *MYCN* haploinsufficiency, which represents the established pathogenic mechanism underlying Feingold syndrome type 1 (FS1). According to the ACMG/ClinGen guidelines for the interpretation of copy number variants, this deletion was classified as pathogenic. Loss of a copy of DDX1 is not related to a specific pathologic disease, however on the other hand, loss of a copy of *MYCNUT* and *MYCNOS* genes play an important role in MYCN expression’s regulation.

Following genetic counseling, parental CMA testing was performed. Analysis of the father revealed the same heterozygous ~342 kb deletion at 2p24.3 (chr2:15,768,340–16,111,204), confirming autosomal dominant inheritance of the pathogenic copy number variant. The paternal phenotype included bilateral clinodactyly and brachymesophalangia; while he completed secondary education, he did not pursue tertiary education, and he is currently employed as a farmer.

At 30^+4^ weeks gestational age, in the scheduled ultrasound, there was increased resistance in the maternal vessels, with the mean pulsatility index (MPI) measured at 1.8, and the fetal growth was estimated under the 5th percentile for gestational age. After this, the patient was advised to undergo a more intensive follow-up, including Doppler ultrasound every three weeks due to a higher risk of developing preeclampsia and fetal growth restriction (FGR). During the follow-up appointments, MPIs of the uterine arteries, umbilical artery, ductus venosus, and middle cerebral artery were measured for fetal distress indications. Moreover, regular daily measurements of arterial blood pressure and weekly urine testing for early detection of proteinuria were recommended.

At 33^+3^ weeks, the patient developed preeclampsia (high blood pressure and proteinuria), and she underwent an immediate caesarean section. She delivered a female newborn weighing 1700 gr, with Apgar scores measured at 9 and 10 at the 1st and 5th minutes, respectively. The ultrasonographic findings were verified, and the newborn had bilateral clinodactyly and thumb hypoplasia, while head circumference was measured below the 5th percentile (28.5 cm). Fortunately, the neonate did not present any gastrointestinal disorders, and no operations were needed.

## 3. Discussion

In this case, a prenatal diagnosis of FS was established based on ultrasound findings of bilateral clinodactyly and microcephaly. The molecular karyotype revealed that the fetus and father carried a heterozygous ~342 kb deletion encompassing MYCN, resulting in MYCN haploinsufficiency, the known pathogenic mechanism for FS1. Tracheoesophageal or duodenal malformations, microcephaly, and developmental delay are classical indications for referring patients with FS1 [7]. However, diagnosis of FS1 can be a challenge in cases with uncommon features and without digestive defects that can draw attention during the anatomy scan test [7]. This favorable phenotype of the disease presents with no gastrointestinal disorders, and no further management is required.

The syndrome is classified into two subtypes: FS type 1 (FS1) is observed as a result of *MYCN* gene variations, specifically at the 2p24 locus that encodes the proto-oncogene protein N-myc [2,3]. The autosomal dominant FS1, or oculodigital–esophageal–duodenal syndrome, manifests as a result of haploinsufficiency of the *MYCN* gene [9]. FS type 2 (FS2), on the other hand, is caused by haploinsufficiency of the 13q region, spanning the MIR17HG gene that encodes the miR17-92 cluster containing six miRNA subgroups (miR17, miR18a, miR19a, miR19b1, miR20a, and miR92) [10]. These genes and their respective proteins play a pivotal role in the early stages of embryogenesis, especially for the development of the skeletal system [5]. The main differences between the two types of FS are displayed in Table 1. A finding worth underlining is the fact that our case of FS1, as with the paternal phenotype, was not associated with gastrointestinal anomalies despite deletions in the *MYCN* gene.

The *MYCN* gene (Mendelian Inheritance in Man MIM number: 164840) is classified under the extended MYC proto-oncogene family. The proteins produced by the MYC genes modulate the expression of a plethora of genes by functioning as transcription factors. These, in turn, are involved in essential cellular processes, including cell proliferation, differentiation, and apoptosis. Additionally, recent evidence indicates target gene-independent roles that include facilitating transcription, termination, and elongation [7]. Initially, MYCN was used as a biomarker in neuroblastoma, as it was present in 20–30% of neuroblastoma patients, and its presence was associated with poorer prognosis [8]. Moreover, Wilms’ tumor, rhabdomyosarcomas, and lung cancers are associated with amplification of the *MYCN* gene [11,12,13,14,15,16,17]. Nevertheless, MYCN and other MYC proteins play a crucial role in organogenesis—specifically, in the development of the fetal heart, kidneys, lungs, brain, and limbs—in addition to their implication in malignant tumors [12,13,14,15,16,17]. For this reason, and according to these expression patterns, heterozygous mutations that result in loss of function of the *MYCN* gene negatively impact fetal development, resulting in FS with multiple congenital abnormalities [7].

In a review published in 2003 by Celli et al. [18], 79 patients from 25 families with FS were reported, and the most common clinical features were presented. Brachymesophalangy was the most common feature (95%), followed by microcephaly (86%), toe syndactyly (80%), short palpebral fissures (57%), learning disability (41%), and gastrointestinal atresia (38%). Cardiac and renal anomalies were less frequent, reported in 14% and 5% of patients, respectively. Hearing loss was reported in 7% of patients [13]. Additionally, Marcelis et al. [19] reported similar data to Celli et al. [18]. after recruiting 77 more patients, complementing the common physical features of FS1. Moreover, short stature was reported in 60% and micrognathia in 32% of patients [14].

The diagnostic criteria for FS, as delineated by Cognet et al. [20], include the presence of three or more of the following basic characteristics: (i) microcephaly, (ii) brachymesophalangy of the second and fifth fingers, (iii) syndactyly of the second and third or fourth and fifth toes, and (iv) gastrointestinal atresia, specifically in the esophagus or duodenum. While postnatal microcephaly remained consistent at three years of age, head circumference was normal at delivery in three instances. All patients exhibited mild-to-moderate intellectual disability, and eight experienced postnatal growth retardation [15].

In a case series by Tedesco et al. [21] that included six distinct families, only four out of eleven patients affected by FS presented with gastrointestinal atresia, while common features comprised digital anomalies—specifically, brachymesophalangy, clinodactyly, and hypoplastic thumbs—as well as almost all showing microcephaly below the third centile [16]. Moreover, Peleg et al. [22] reported a rare expression of FS1; they described a pedigree combining familial congenital absence of the flexor pollicis longus tendon (CAFPL) as an attribute of FS1. Molecular investigations after whole-exome sequencing of five affected family members across three generations identified a new *MYCN* gene mutation (c.1171C>T; p.Arg391Cys). To date, no documented association between *MYCN* variants and either solitary or syndromic CAFPL has been reported. CAFPL is considered to be an uncommon hand abnormality. Most occurrences are sporadic, and no genetic variations have been identified in association with this anomaly [21].

While gastrointestinal atresia is unique in FS1, the skeletal system is the most affected system in FS2. Particular features—such as short stature, microcephaly, brachymesophalangia, and fifth-finger clinodactyly—can be observed in these patients. The role of the *MIR17HG* gene is paramount for the proliferation of osteoblasts, as well as for their differentiation, as demonstrated in experiments with mice [5]. Homozygous deletions of the *MIR17HG* gene in mice were reported to cause perinatal mortality. Nevertheless, the heterozygous deletions resulted in skeletal developmental disorders and brachymesophalangia [10]. Brachymesophalangia was seen in 100% of the studied patients (16/16), short stature in 81% (13/16), and fifth-finger clinodactyly in 68% (11/16).

The level of cognitive impairment associated with FS is typically mild, with overt intellectual disability considered to represent rare cases. Burnside et al. [5], based on results from a case series of six individuals diagnosed with FS, noticed that large deletions were linked with more severe intellectual disability, likely due to haploinsufficiency of other genes. From the same cohort of patients, other atypical features observed included structural brain abnormalities, genital abnormalities, motor delay, and radioulnar synostosis [22]. The overarching phenotypes were attributable to FS1 due to haploinsufficiency of MYCN. Clinical features of individuals with deletions encompassing numerous additional genes are likely to be more complex and/or more severe than typical FS1 features. The contribution of specific genes to these additional features is thus far undefined. A number of deleted *OMIM* genes mentioned in this study have autosomal dominant inheritance, but none other than SOX11 and MYCN are known to have haploinsufficiency effects.

Preeclampsia, placental abruption, and fetal growth restriction (FGR) are collectively referred to as placental ischemic disease (PID). This classification arises from their common features of uteroplacental malperfusion, chronic hypoxia, and ischemia of the placenta. Inadequate trophoblast invasion and incomplete remodeling of the spiral arteries during placentation represent the main pathophysiological pathway of PID [23]. Preeclampsia is defined as hypertension and proteinuria occurring later than 20 weeks of gestation in a previously normotensive patient, based on the American College of Obstetrics and Gynecology (ACOG) guidelines [24]. Preeclampsia has also been linked with long-term sequelae for maternal health [25]. Based on the International Society of Ultrasound in Obstetrics and Gynecology (ISUOG) guidelines, FGR is divided into early and late forms based on the timepoint of 32 weeks. The major criteria include the EFW and abdominal circumference (AC) below the third centile in both instances. In the case of early FGR, a pulse index (PI) of the umbilical artery (UA) above the 95th centile is also included. Contributory factors include AC/EFW 95th centile and UA PI > 95th centile in the early form; and AC/EFW < 10th centile, AC/EFW crossing centiles > two quartiles on growth centiles, and cerebroplacental ratio (CPR) 95th centile in the late form [26].

Based on published data from other FS cases, our patient presented skeletal developmental abnormalities (bilateral clinodactyly) and microcephaly that were present simultaneously (Figure 1 and Figure 2). Fortunately, in our case, no gastrointestinal disorders were present. However, to the best of our knowledge, our case is the first report of the simultaneous occurrence of FS and placental ischemic disease (FGR and preeclampsia). This extremely rare concurrence could be a coincidence, or it could be the result of an unknown molecular pathway linking *MYCN* genes with placental ischemic disease. After an extensive search of available published cases, only one case of FS complicated with FGR was reported in 2007 [27]. More case series reporting the obstetric outcomes of fetuses diagnosed with FS will be necessary to elucidate the possible link between FS and placental ischemic disease.

## Figures and Tables

**Table 1:** Main differences between the two types of Feingold syndrome.

Feingold Syndrome Type	Type 1 (FS1)	Type 2 (FS2)
**Chromosome**	2p24 locus	13q region
**Gene Variations**	*MYCN* gene	*MIR17HG* gene
**Protein**	N-myc	miR17-92 cluster

## Data Availability

The original contributions presented in this study are included in the article. Further inquiries can be directed to the corresponding author(s).
